# Transformed follicular lymphoma with laryngeal edema requiring tracheal intubation after tisagenlecleucel treatment: A case report

**DOI:** 10.1097/MD.0000000000039630

**Published:** 2024-09-06

**Authors:** Ryoma Shimazu, Nobuhiko Nakamura, Takayuki Goto, Yuto Kaneda, Yoshikazu Ikoma, Takuro Matsumoto, Hiroshi Nakamura, Nobuhiro Kanemura, Masahito Shimizu

**Affiliations:** a Department of Hematology and Infectious Disease, Gifu University Hospital, Gifu, Japan.

**Keywords:** cytokine release syndrome, tisagenlecleucel, transformed follicular lymphoma

## Abstract

**Rationale:**

Cytokine release syndrome (CRS) is a common adverse event of chimeric antigen receptor T (CAR-T) cell therapy. CRS is generally a systemic inflammatory reaction, but in rare cases, it can occur in specific body areas and is referred to as “local CRS (L-CRS).” A case of laryngeal edema due to L-CRS that required tracheal intubation because of the lack of response to tocilizumab (TCZ) and dexamethasone (DEX) is reported.

**Patient concerns:**

A 67-year-old woman with relapsed transformed follicular lymphoma was treated with CAR-T cell therapy. Although she had been given TCZ and DEX for CRS, neck swelling appeared on day 4 after infusion.

**Diagnoses:**

Laryngoscopy showed severe laryngeal edema, which was presumed to be due to L-CRS, since there were no other apparent triggers based on history, physical examination, and computed tomography.

**Interventions:**

Tracheal intubation was performed because of the risk of upper airway obstruction. Ultimately, 4 doses of tocilizumab (8 mg/kg) and 6 doses of dexamethasone (10 mg/body) were required to improve the L-CRS.

**Outcomes:**

On day 7, laryngeal edema improved, and the patient could be extubated.

**Lessons:**

The lessons from this case are, first, that CAR-T cell therapy may induce laryngeal edema in L-CRS. Second, TCZ alone may be ineffective in cervical L-CRS. Third, TCZ, as well as DEX, may be inadequate. In such cases, we should recognize L-CRS and manage it early because it may eventually progress to laryngeal edema that requires securing the airway.

## 1. Introduction

The prognosis of patients with relapsed or refractory diffuse large B-cell lymphoma (DLBCL) is generally poor.^[1,2]^ Chimeric antigen receptor T (CAR-T) cells targeting CD19 have shown clear efficacy against B-cell acute lymphoblastic leukemia, follicular lymphoma (FL), and DLBCL refractory to standard immunochemotherapy.^[3,4]^ However, specific adverse events such as cytokine release syndrome (CRS), immune effector cell-associated neurotoxicity syndrome, and B-cell aplasia are commonly reported with CAR-T cell therapy.^[5,6]^ CRS is caused by the release of cytokines from CAR-T cells, which activate monocytes and macrophages and lead to the development of systemic inflammation. However, in rare cases, CRS can develop in specific body areas, referred to as “local CRS (L-CRS).”^[7]^

This report describes a case of laryngeal edema due to L-CRS of the neck that led to tracheal intubation. Written, informed consent was obtained from the patient to publish this case report and accompanying images.

## 2. Case presentation

A 67-year-old woman who had been diagnosed with FL at age 44 years and had relapsed 3 times since then complained of anterior chest pain. Computed tomography (CT) showed tumors in the mediastinum and stomach, and biopsies from both lesions led to the diagnosis of DLBCL (CD19^+^, CD20^+^), suggesting transformation from FL. Positron emission tomography (PET)-CT showed no abnormal FDG uptake in the bone marrow, and no abnormal lymphocytes were noted in the peripheral blood. After the first cycle of chemotherapy, consisting of gemcitabine, carboplatin, dexamethasone, and rituximab,^[8]^ the tumor size had decreased by only about 40%, and it was determined that it was not sufficiently effective. Therefore, it was thought CAR-T that therapy was indicated. Therefore, after the second cycle of gemcitabine, carboplatin, dexamethasone, and rituximab therapy, lymphocyte collection was performed (CD3-positive cell count 3.1 × 10^9^). Subsequently, 2 cycles of chemotherapy consisting of polatuzumab vedotin, bendamustine, and rituximab were administered as bridging therapy, and complete remission was then confirmed by PET-CT and upper gastrointestinal endoscopy.

**Figure 1.**
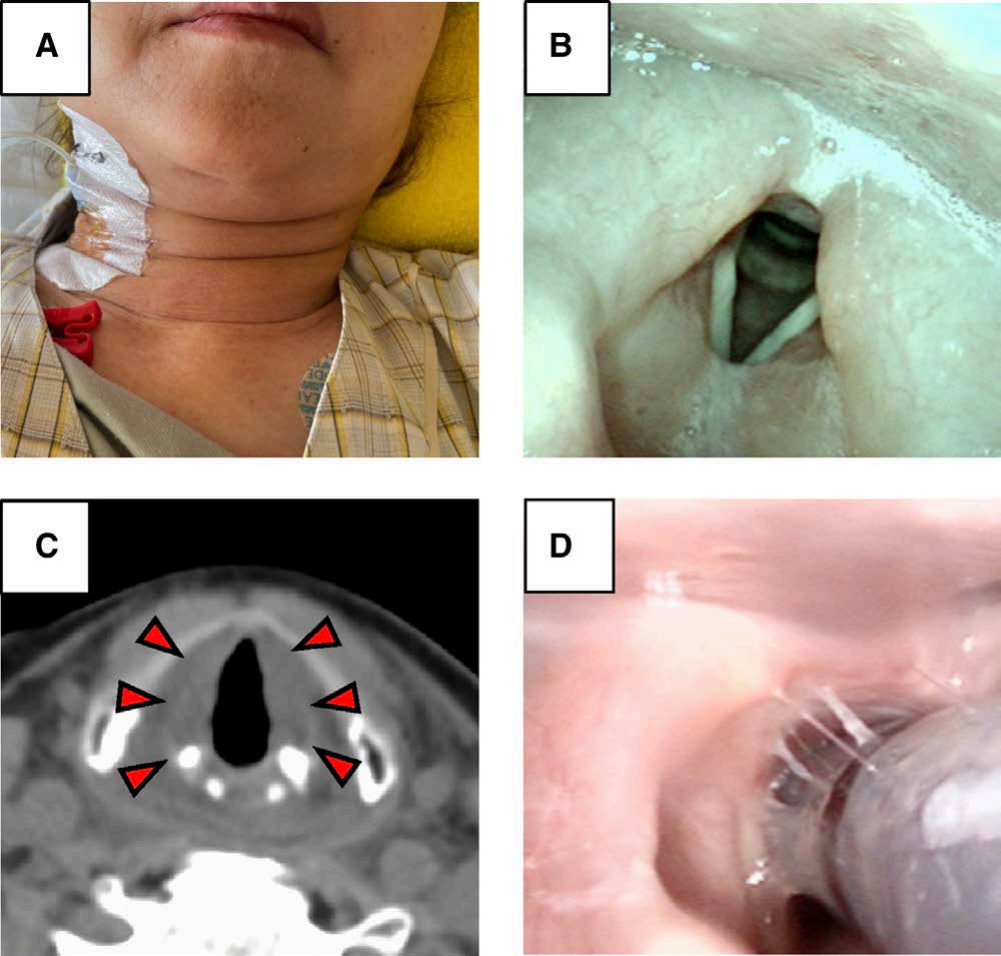
(A) Photograph of the face and neck on day 4 after CAR-T cell administration. Swelling of the face and neck is observed. (B) Image from laryngoscopy. Marked laryngeal edema can be seen. (C) Neck CT before tracheal intubation. Marked laryngeal edema can be seen (red arrows). (D) Image from laryngoscopy. Laryngeal edema is improving.

**Figure 2.**
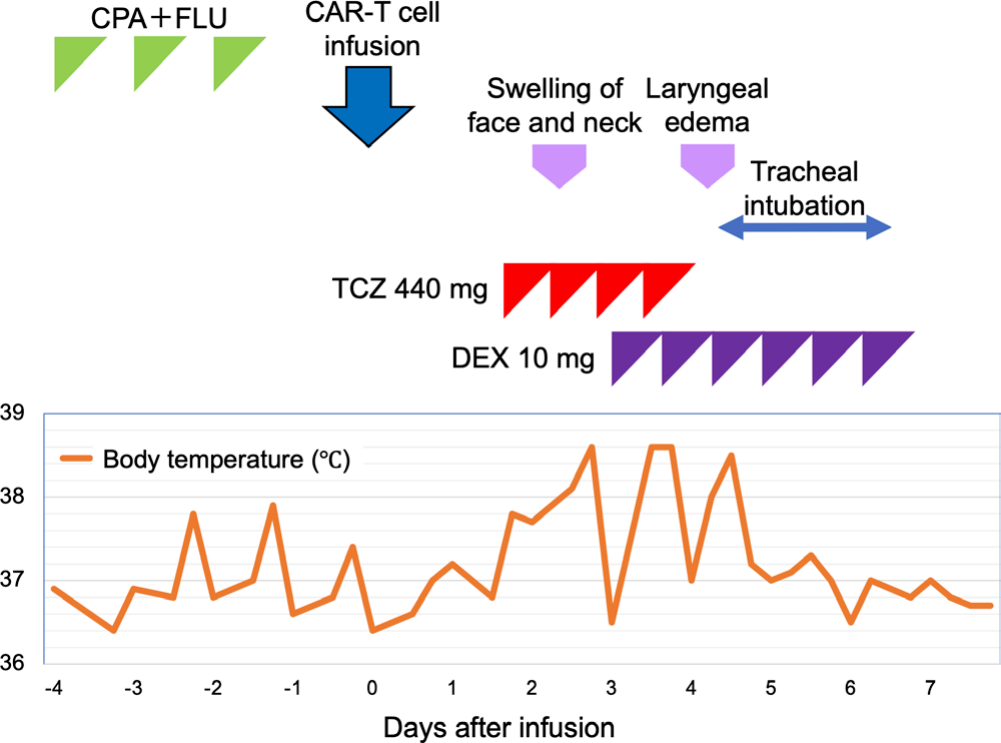
This patient’s clinical course of treatment after admission to the hospital. CAR-T = chimeric antigen receptor-T, CPA = cyclophosphamide, DEX = dexamethasone, FLU = fludarabine, TCZ = tocilizumab.

The patient received lymphocyte-depleting chemotherapy with cyclophosphamide and fludarabine, followed by tisagenlecleucel infusion. The day after receiving CAR-T cell infusion (day 1), the patient developed a fever of 38 °C and was given TCZ 8 mg/kg with a diagnosis of CRS grade 1. However, during the night of day 2, despite administration of TCZ, the patient developed decreased oxygenation and edema of the neck and face. Therefore, TCZ and dexamethasone (DEX: 10 mg) were administered 3 times each, but no improvement was observed (Fig. 1A). The patient also presented with sore throat and hoarseness, so laryngoscopy was performed on day 4, which showed marked laryngeal edema (Fig. 1B). Plain CT on the same day also showed significant laryngeal edema (Fig. 1C), and the patient was intubated due to concern about airway obstruction. Since there was no history of angioedema-inducing medications and no skin rash or increased eosinophils suggestive of allergy, the laryngeal edema was assumed to be due to L-CRS. After 3 additional doses of DEX were administered under intubation, the laryngeal edema improved on day 7 (Fig. 1D), and the patient was extubated. The patient’s subsequent progress was good, and she was discharged on day 24 (Fig. 2).

## 3. Discussion

A case of laryngeal edema due to L-CRS of the neck that led to tracheal intubation was described. This case highlights 3 findings related to L-CRS. First, CAR-T cell therapy can induce laryngeal edema in L-CRS. Second, TCZ alone may be ineffective for cervical L-CRS. Third, TCZ, as well as DEX, may be inadequate, and in such cases, L-CRS may eventually progress to laryngeal edema that requires securing the airway.

**Figure 3.**
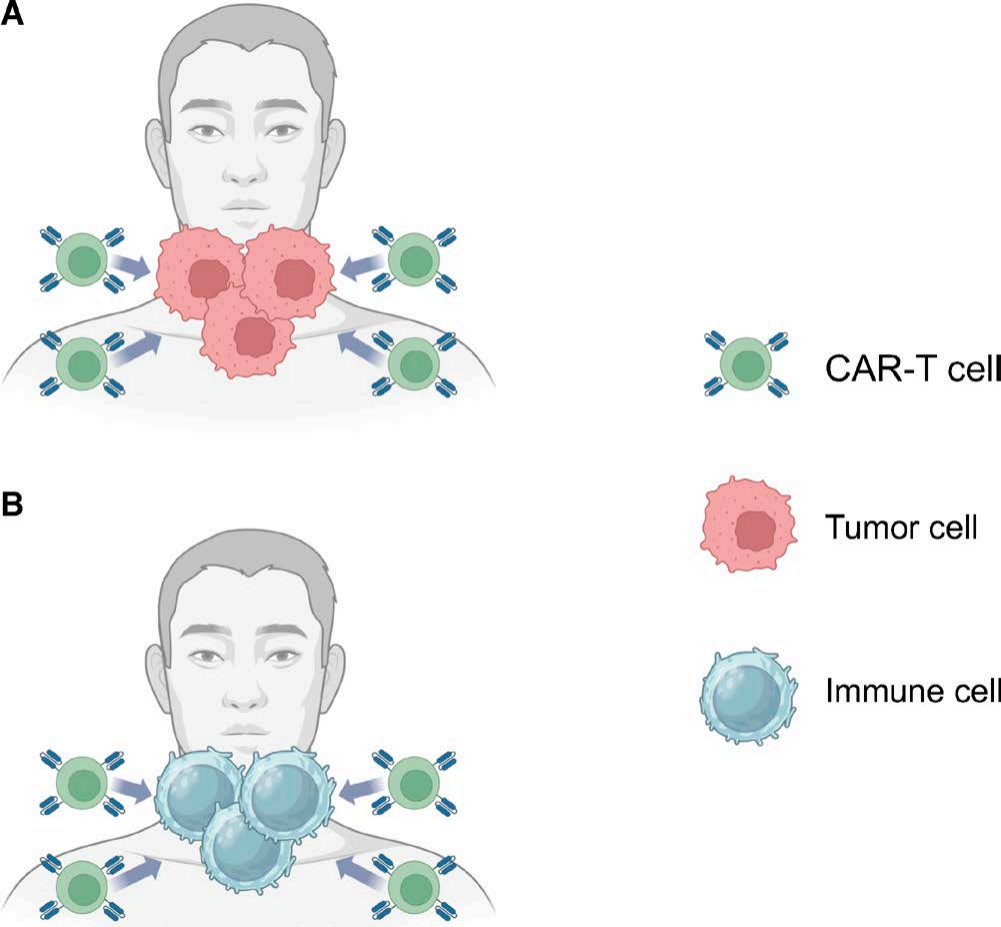
(A) The model of L-CRS in the presence of a tumor mass, as in malignant lymphoma. After CAR-T cell infusion, the cells proliferate within the tumor mass. Subsequently, CAR-T cells induce L-CRS as a localized inflammatory response. Once the tumor cells disappear locally, CAR-T cells and cytokines migrate systemically, producing systemic CRS. (B) The model of L-CRS in the absence of an obvious tumor mass, as in B-ALL. After CAR-T cell infusion, the cells proliferate in response to tumor cells distributed throughout the body, and they attack tumor cells. Then, CAR-T cells recognize and redistribute to sites where tumor cells may be present or cells that express the same antigens as the tumor cells. B-ALL = B-cell acute lymphoblastic leukemia, CAR-T = chimeric antigen receptor-T.

L-CRS is a rare pathology that differs from usual systemic CRS. As of August 2023, only 7 cases of cervical L-CRS had been reported, 3 of which had lymphomas.^[9–14]^ The following mechanisms of L-CRS have been discussed, but not fully elucidated. One mechanism of L-CRS is assumed to be due to localized lymphoma lesions (Fig. 3A).^[7]^ After infusion of CAR-T cells, the cells increase locally within the tumor mass, resulting in L-CRS as a strong localized inflammatory response. After the tumor cells disappear locally, these proliferated CAR-T cells and cytokines migrate systemically, producing systemic CRS. The other mechanism of L-CRS is assumed when there is no obvious local tumor mass, as in B-cell acute lymphoblastic leukemia (Fig. 3B).^[10]^ After CAR-T cell infusion, they increase in response to tumor cells distributed throughout the body, attack tumor cells, and release large amounts of cytokines. After eliminating tumor cells at most sites in the body, CAR-T cells may recognize and redistribute to sites where tumor cells may be present (e.g., the neck, where lymphoid tissue is abundant, and tumor antigens may be present) or cells expressing the same antigens as the tumor cells, leading to severe inflammation. After that, the CRS would improve. The present patient had a history of recurrence in the bilateral tonsils at the time of the second FL relapse at age 52 years. No cervical lesions were present at the time when final recurrence transformed to DLBCL, but the possibility that lymphoma lesions not noted on PET-CT were located in the neck, including the tonsils, cannot be ruled out. Of the 4 cases of malignant lymphoma in the present and previous case reports,^[9,12]^ 1 had cervical lesions at the time of CAR-T cell infusion, and 2 had a history of cervical lesions. These reports and the present findings suggest that the presence or history of cervical lesions may be a risk factor for laryngeal edema.

If systemic CRS appears during CAR-T cell therapy, it is recommended that TCZ, a monoclonal antibody against the interleukin-6 receptor, be used as soon as possible.^[15]^ However, the effect of TCZ on L-CRS may be limited. In the present case, TCZ was administered a total of 4 times, but life-threatening laryngeal edema appeared, and tracheal intubation was required. As in the present case, most previous cases (6/7 cases) were treated with the addition of DEX, because TCZ alone did not improve laryngeal edema. These findings suggest that TCZ alone may not adequately control the progression to severe, life-threatening laryngeal edema associated with L-CRS.

Furthermore, DEX is also sometimes ineffective for L-CRS. In the present case, despite 3 10-mg doses of DEX administered during the night on day 2, laryngeal edema continued to develop, and tracheal intubation was eventually required. However, as in most previous reports, cervical edema improved within a few hours of DEX administration,^[9,11–13]^ and response to DEX is generally considered good in L-CRS. Factors related to a poor DEX response of S-CRS after CAR-T therapy include high tumor burden, rapid progression of CRS, delay in DEX administration, and co-existing infections.^[16,17]^ However, none of these factors was involved in the present case; therefore, it is unclear what caused the poor response to DEX in the present case. Thus, it is important that clinicians pay close attention to neck swelling, which is a precursor to laryngeal edema, when administering CAR-T cell therapy. If cervical swelling is observed, the larynx should be aggressively evaluated by laryngoscopy or other means to detect and respond to exacerbation of life-threatening laryngeal edema at an early stage. Furthermore, in the event of severe laryngeal edema, it is also important to not hesitate to perform tracheal intubation for airway management.

This study did have some limitations. First, since this study was conducted in a single university hospital, one must be cautious about generalizing the results. Analysis of cases in different healthcare settings and geographical locations would be useful to confirm the external validity of the results. Second, since this study reports on a patient treated with tisagenlecleucel, it is not clear whether the findings in this case could be applied to other CAR-T cell therapies such as axicabtagene ciloleucel and lisocabtagene maraleucel, and analysis of further cases is needed to clarify this. Third, in this study, DEX was administered at a dose of 10 mg after the appearance of neck swelling, but further increases in dose or frequency of administration may not necessarily result in the same laryngeal edema as in the present case. Further research is also needed on the appropriate dose and frequency of administration of DEX when neck swelling appears.

## 4. Conclusion

Clinical lessons from this case and previous studies include: first, CAR-T cell therapy may induce laryngeal edema in L-CRS; second, TCZ alone may be ineffective in cervical L-CRS; and third, TCZ, as well as DEX, may be inadequate. Therefore, clinicians must pay close attention to neck swelling when administering CAR-T cell therapy, and if cervical swelling is observed, the larynx should be aggressively evaluated by laryngoscopy or other means to detect exacerbation of life-threatening laryngeal edema at an early stage. In the event of severe laryngeal edema, it is also important to not hesitate to perform tracheal intubation for airway management.

## Acknowledgments

The authors would like to thank all staff of the Department of Hematology and Infectious Diseases at Gifu University Hospital for their helpful advice in preparing this manuscript.

## Author contributions

**Conceptualization:** Nobuhiko Nakamura.

**Investigation:** Ryoma Shimazu, Nobuhiko Nakamura, Takayuki Goto, Yuto Kaneda, Yoshikazu Ikoma, Takuro Matsumoto, Hiroshi Nakamura, Nobuhiro Kanemura, Masahito Shimizu.

**Supervision:** Nobuhiro Kanemura, Masahito Shimizu.

**Writing – original draft:** Ryoma Shimazu.

**Writing – review & editing:** Nobuhiko Nakamura.
